# Gastroprotective Action of Adiponectin Against Gastric Mucosal Injury Induced by Ischemia and Reperfusion—Involvement of Nitric Oxide, Sensory Afferent Nerves, and Anti-Inflammatory Mediators

**DOI:** 10.3390/ijms27062827

**Published:** 2026-03-20

**Authors:** Sławomir Kwiecien, Aleksandra Szlachcic, Dagmara Wojcik-Grzybek, Zbigniew Sliwowski, Malgorzata Strzalka, Urszula Szczyrk, Agata Ptak-Belowska, Tomasz Brzozowski

**Affiliations:** Department of Physiology, Faculty of Medicine, Jagiellonian University Medical College, 31-531 Cracow, Poland; skwiecien@cm-uj.krakow.pl (S.K.); aleksandra.szlachcic@uj.edu.pl (A.S.); dagmara1.wojcik@uj.edu.pl (D.W.-G.); zbigniew.sliwowski@uj.edu.pl (Z.S.); malgorzata.strzalka@uj.edu.pl (M.S.); urszula.szczyrk@uj.edu.pl (U.S.); agata.ptak-belowska@uj.edu.pl (A.P.-B.)

**Keywords:** adiponectin, ischemia and reperfusion, nitric oxide, capsaicin, lipid peroxidation

## Abstract

Adiponectin is adipokine exhibiting beneficial metabolic action through lipid and carbohydrate metabolism stimulation, as well as anti-inflammatory action. We have determined the role of adiponectin in gastroprotection against the formation of acute gastric lesions induced by ischemia–reperfusion (I/R). Gastric lesions evoked by I/R are a serious clinical entity; however, the participation of reactive oxygen species (ROS) and lipid peroxidation products and the involvement of nitric oxide (NO), neuropeptides released from sensory afferent nerves, and the hormone gastrin in the potential gastroprotective action of adiponectin remains unknown. Therefore, we determined the interplay between capsaicin-sensitive afferent nerves, the NO/NOS system, lipid peroxidation products, and the expression of pro-inflammatory and antioxidative factors in the gastroprotective action of adiponectin against gastric I/R. injury. Wistar rats was administered with adiponectin in graded doses (1–40 μg/kg i.v.) with or without: (a) blockade of nitric oxide (NO) activity by L-nitro-L-arginine (L-NNA) and (b) deactivation of sensory nerves by capsaicin (125 mg/kg s.c. 10 days before experiment conduction). They were then exposed to 30 min of ischemia by clamping of the celiac artery followed by 3 h of reperfusion after clamp release. After 3 h, the rats were euthanized with pentobarbital and their gastric blood flow (GBF) was determined by laser Doppler flowmetry, their blood was withdrawn to assess plasma gastrin levels, and the area of gastric lesions was measured by planimetry. Gastric biopsy samples were excised to determine gastric mucosal levels of malondialdehyde (MDA) and 4-hydroxynonenal (4-HNE). In separate groups of animals with chronic gastric fistula, the effect of adiponectin on gastric acid secretion was determined. Adiponectin dose-dependently reduced the gastric lesions induced by I/R and this effect was accompanied by an increase in GBF. Blockade of NO-synthase with L-NNA (20 mg/kg i.p.) reversed the protective effect and the rise in GBF induced by this adipokine, and both these effects were restored when L-arginine was added to L-NNA. Capsaicin denervation also impeded the beneficial action of adiponectin in rats, but these effects were in part restored when exogenous CGRP was combined with adiponectin. Adiponectin dose-dependently decreased gastric acid secretion, the expression of mRNA for pro-inflammatory cytokines, and MDA plus 4-HNE content, while significantly increasing SOD, GSH and plasma gastrin increments. We conclude that adiponectin exerts gastroprotection against I/R-induced gastric lesions, through mechanisms involving NO and neuropeptides such as CGRP being released from sensory nerves, a decrease in lipid peroxidation (MDA+4-HNE), an increase of antioxidative factors (SOD, GSH), and the inhibition of gastric acid secretion.

## 1. Introduction

Adiponectin, produced by adipocytes, exhibits beneficial metabolic action through lipid and carbohydrate metabolism stimulation and exerts anti-inflammatory properties by inhibiting tumor necrosis factor alpha (TNF-α) [1]. The important protective role of adiponectin in the development of metabolic syndrome and diabetes has been documented [2,3], but its significance in the mechanism of gastroprotection, especially under of ischemia–reperfusion (I/R) conditions, has been little studied. Acute gastric mucosal lesions comprise an important clinical problem. The integrity of the gastric mucosa depends on a variety of factors. Gastric microcirculation, regulated by nitric oxide (NO), plays a vital role in the maintenance of gastric mucosal barrier integrity and gastroprotection [4]. Sensory nerves, containing and releasing vasodilator peptides, such as calcitonin gene-related peptide (CGRP), are involved in the regulation of gastric mucosa blood flow. Therefore, the functional ablation of sensory nerves by high doses of capsaicin provides the opportunity to determine their role in the mechanism of gastric protection [5]. NO, which cooperates with CGRP in the maintenance of gastric integrity and gastroprotection [5,6], is produced and released from the vascular endothelium and sensory nerve endings via the enzymatic activity of NO synthase (NOS), which was inhibited under experimental conditions by the NOS inhibitor N-nitro-L-arginine (L-NNA) [7]. The pathogenesis of I/R injury depends upon the large amount of reactive oxygen species (ROS) produced in the gastric mucosa. An ischemic episode in the gastric mucosa leads to the excessive generation of ROS during reperfusion, when the availability of oxygen is increased and the superoxide radical anion (O_2_^•−^) is produced. The superoxide radical anion (O_2_^•−^) reacts with cellular lipids, leading to the formation of lipid peroxides, which are metabolized to malondialdehyde (MDA) and 4-hydroxynonenal (4-HNE). The peroxidation of lipids in cellular membranes affects their physiology (changes in permeability and polarization; membrane becomes stiffer). Moreover, MDA reacts with the amine moiety of protein, making Schiff bases (amino aldehydes) and, in this way, disturbs the action of cellular pumps, built by proteins. Superoxide radical anions (O_2_^•−^) can be scavenged by antioxidative systems, involving SOD and GSH [5,6].

Previous studies documented that obesity, caused by a high fat diet, resulted in the enhancement of lipid peroxidation and the impairment of SOD activity. These parameters of oxidative stress were corrected by adiponectin [8]. These changes may be partially associated with an imbalance of leptin and adiponectin. Leptin up-regulates pro-inflammatory cytokines, amplifying oxidative stress. Adiponectin reveals opposite, i.e., antioxidative, properties, which counteract the inflammatory response and the production of oxygen free radicals [9].

However, the participation of ROS generation and lipid peroxidation products and the involvement of NO and sensory afferent nerves in the potential gastroprotective action of adiponectin remains unknown. Therefore, the aim of our present investigations was to provide explanatory detail on the interplay between adiponectin and the above-mentioned factors.

## 2. Results

### 2.1. Gastric Acid Secretion

Table 1 presents the effect of administration of adiponectin, applied i.v. in graded doses ranging from 1 µg/kg up to 40 µg/kg, on gastric H^+^ concentration in GF rats. The pretreatment with adiponectin caused a dose-dependent reduction in gastric acid output starting from the dose of 5 µg/kg and the maximal inhibition of gastric acid secretion was observed when adiponectin was injected in a dose of 40 µg/kg (i.v.).

### 2.2. Gastric Lesions, Gastrin Plasma Level, and Gastric Blood Flow (GBF)

**Figure 1:**
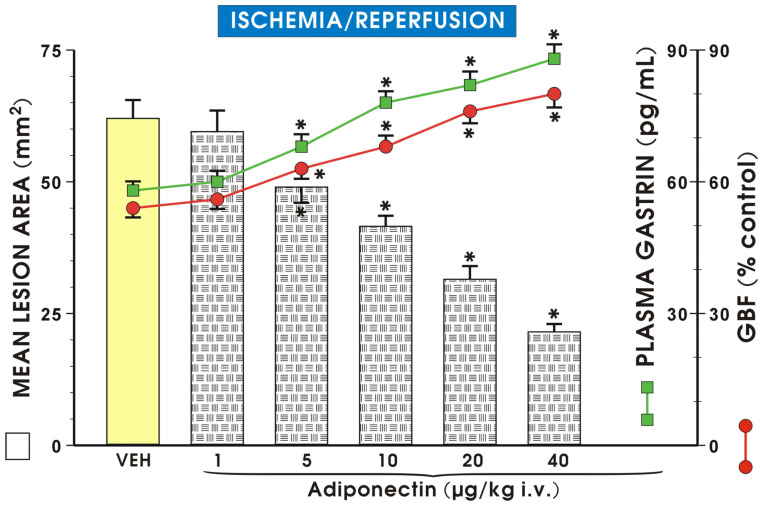
Mean area of gastric lesions (mm^2^), plasma gastrin concentration (pg/mL) and gastric blood flow (GBF) in rats exposed to ischemia (30 min) followed by 3 h of reperfusion (I/R) without or with intravenous (i.v.) pretreatment with adiponectin, applied in graded doses from 1 to 40 µg/kg. Results are mean ± SEM of 8–10 rats. Asterisk (*) indicates a significant change compared with the values obtained in rats exposed to I/R alone (placebo group, VEH = vehicle).

Figure 1 shows the effect of administration of vehicle or adiponectin applied i.v., in graded doses ranging from 1 µg/kg up to 40 µg/kg on the mean lesion area of gastric lesions induced by I/R and the accompanying changes in the GBF and gastrin plasma levels. Pretreatment with adiponectin dose-dependently reduced the mean area of I/R lesions and significantly raised GBF and plasma gastrin concentration. The maximal protective effect was exerted by adiponectin injected in a dose of 40 µg/kg (i.v.) and this dose of adiponectin has been used in subsequent experiments.

**Figure 2:**
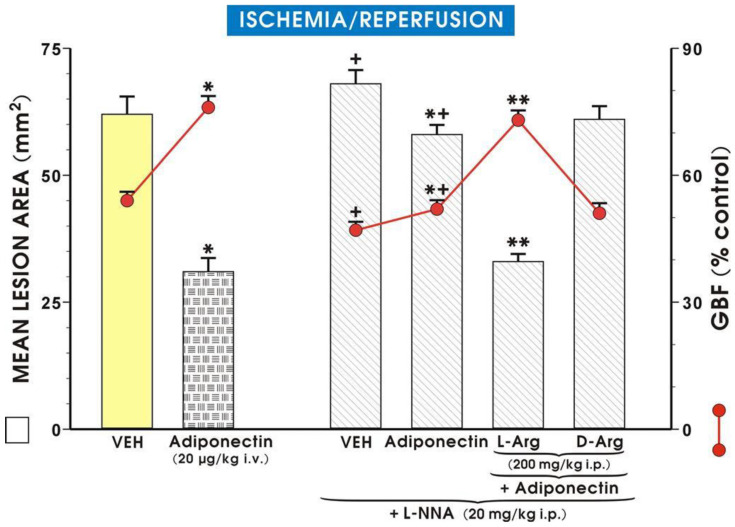
Mean area of gastric lesions (mm^2^) and gastric blood flow (GBF) in rats exposed to 30 min of ischemia, followed by 3 h of reperfusion (I/R) without or with intravenous (i.v.) pretreatment with adiponectin (20 µg/kg) applied alone or given in combination with L-NNA (20 mg/kg i.p.) or with intragastric (i.g.) administration with L-arginine (200 mg/kg), or D-arginine (200 mg/kg), added to L-NNA. Results are mean ± SEM of 8–10 rats. Asterisk (*) indicates a significant change compared with the respective values obtained in rats exposed to I/R alone (placebo group, VEH = vehicle). Cross (+) indicates a significant change compared with the respective values obtained in rats exposed to VEH without treatment with L-NNA + I/R. Asterisk and cross (*+) indicate a significant change compared with the value obtained in rats pretreated with L-NNA. Double asterisks (**) indicate a significant change compared with the value obtained in rats with L-NNA combined with adiponectin against I/R.

As shown in Figure 1, the intact gastric mucosa did not exhibit any macroscopic lesions and the GBF in this mucosa was accepted as the control value (100%). Following I/R, numerous acute gastric mucosal lesions occurred, while GBF was reduced to about 60% of the control value. Pretreatment with adiponectin (1 µg/kg i.v.) failed to affect the area of I/R lesions, but with higher doses of adiponectin (5 µg/kg and 40 µg/kg i.v.) a significant reduction in the area of gastric lesions was observed and these effects were accompanied by a significant increase in GBF compared to control rats pretreated with vehicle (*p* < 0.05). Administration of adiponectin (from 1 µg/kg to 40 µg/kg) resulted in a significant dose-dependent rise in plasma gastrin level (*p* < 0.05), compared with the value of plasma gastrin in vehicle control rats. Figure 2 shows that administration of L-NNA significantly increased the area of I/R-induced gastric lesions and this effect was accompanied by a significant decrease in GBF compared with that obtained in rats with I/R alone (*p* < 0.05).

**Figure 3:**
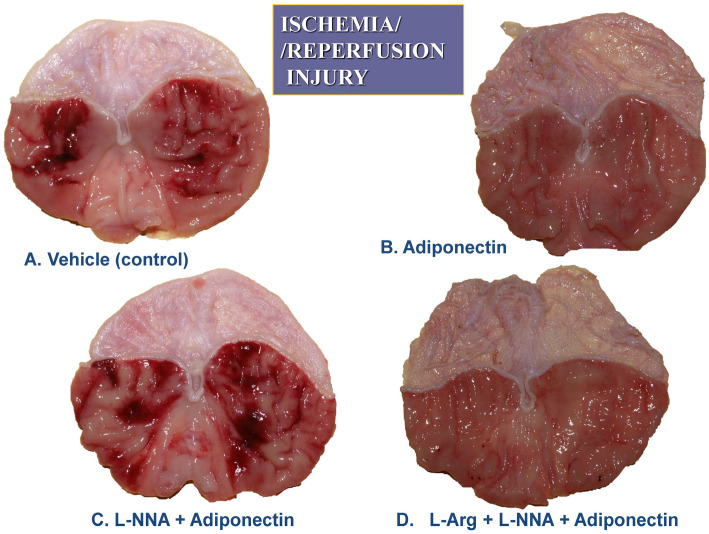
(**A**–**D**). The representative appearance of the rat pretreated with vehicle (control) and 30 min later exposed to 30 min of ischemia, followed by 3 h of reperfusion (I/R) shows the presence of multiple lesions observed in the oxyntic part of the gastric mucosa (**A**). In a rat pretreated with adiponectin (20 µg/kg) and subsequently exposed to I/R, the number of gastric mucosal erosions is reduced (**B**). The number of I/R-induced gastric lesions is markedly increased in the gastric mucosae of animals wherein L-NNA (20 mg/kg) was given in combination with adiponectin (20 µg/kg) (**C**), compared with that assessed in rats wherein adiponectin was applied alone in the I/R model (**B**). The number of I/R-induced gastric mucosal erosions was clearly diminished in the gastric mucosa pretreated with L-arginine (200 mg/kg) with L-NNA (20 mg/kg) and adiponectin (20 µg/kg) (**D**).

The combination of L-NNA (20 mg/kg i.p.) with adiponectin (20 µg/kg i.v.) significantly increased the area of I/R-induced gastric lesions and produced a significant drop in GBF compared to values observed in adiponectin-treated rats without L-NNA administration (*p* < 0.05) (Figure 2). The combined treatment of L-arginine (200 mg/kg i.g.) with L-NNA (20 mg/kg i.p.) in rats administered adiponectin (20 µg/kg i.v.) restored the mean area of gastric lesions and the GBF to the values observed in rats pretreated with adiponectin and subsequently exposed to I/R. In contrast, when D-arginine (200 mg/kg i.g.) was used instead L-arginine, the area of gastric lesions and the accompanying alterations in GBF were similar to those observed in rats given the combination of L-NNA and adiponectin (Figure 2). Figure 3 shows the macroscopic appearance of the gastric mucosa of a rat pretreated with vehicle (control) and 30 min later exposed to 30 min of ischemia followed by 3 h of reperfusion (A).

**Figure 4:**
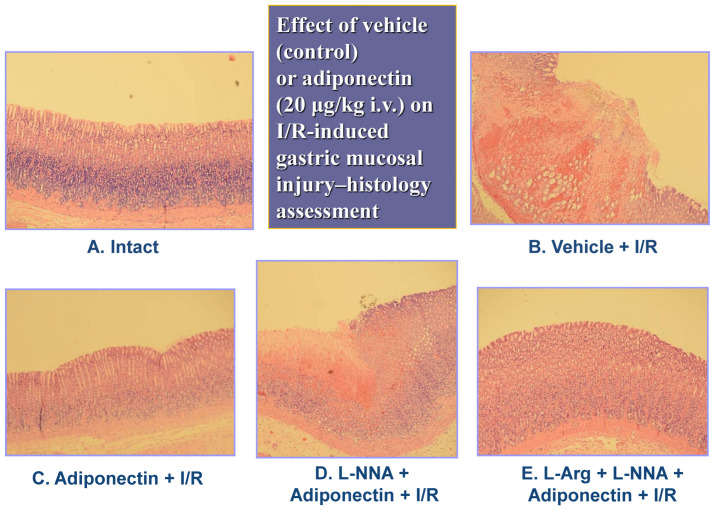
(**A**–**E**). The representative microscopic appearance of the gastric mucosa of an intact rat (**A**) or a rat pretreated with vehicle (control) and 30 min later exposed to 30 min of ischemia, followed by 3 h of reperfusion (I/R). In the vehicle-pretreated control rats exposed to I/R, mucosal damage, as reflected by the degeneration of the surface epithelium and connective tissue of the oxyntic mucosa as well as vascular congestion, is observed (**B**). In a rat pretreated with adiponectin (20 µg/kg) and subsequently exposed to I/R, the epithelial damage was superficial and mild degeneration is observed, but the epithelial cellular lining and deeper layers of the glandular mucosa were diminished, indicating partial preservation of the mucosal structure (**C**). In the gastric mucosae of animals in which L-NNA (20 mg/kg) was given with combination with adiponectin (20 µg/kg) (**D**), the epithelial cells show severe damage. This abnormal histopathological pattern observed in the gastric mucosa in the case of L-NNA addition is improved in the I/R model pretreated with L-arginine (200 mg/kg) with L-NNA (20 mg/kg) and adiponectin (20 µg/kg), due to partial restoration of the glandular structure (**E**). Magnification x20.

The exposure of the gastric mucosa to vehicle applied i.v. 30 min prior to I/R resulted in the presence of multiple lesions observed in the oxyntic part of the gastric mucosa (A). In a rat pretreated with adiponectin (20 µg/kg) and subsequently exposed to I/R, the severity of gastric mucosal erosions was reduced (Figure 3B). The number and area of I/R-induced gastric lesions were markedly increased in the gastric mucosa of an animal pretreated with L-NNA (20 mg/kg), given in combination with adiponectin (20 µg/kg), compared with that assessed in rats for which adiponectin was applied alone for I/R injury (Figure 3C vs. Figure 3B). The number and area of I/R-induced gastric mucosal erosions were clearly diminished in the gastric mucosa pretreated with the combination of L-arginine (200 mg/kg) and L-NNA (20 mg/kg) which later received adiponectin (20 µg/kg) (Figure 3D vs. Figure 3C). Figure 4A,B demonstrates a microscopic view of the gastric mucosa of an intact rat (A) or a rat pretreated with vehicle (control) and 30 min later exposed to 30 min of ischemia followed by 3 h of reperfusion (I/R) (B).

**Figure 5:**
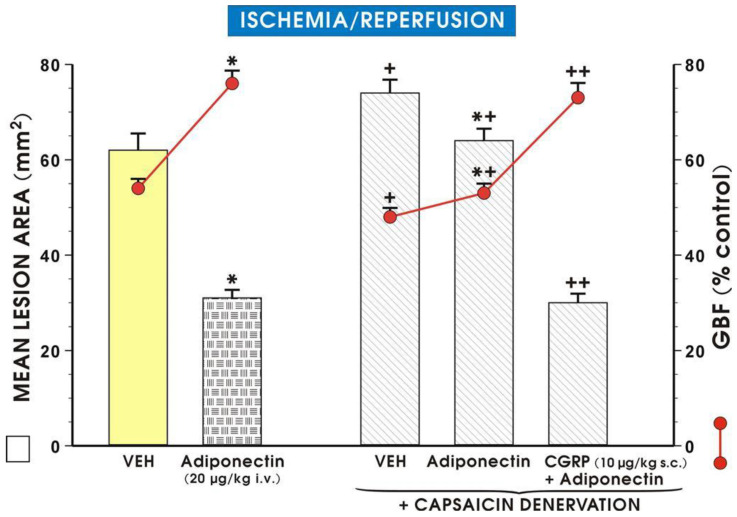
Mean area of gastric lesions (mm^2^) and gastric blood flow (GBF) in rats exposed to 30 min of ischemia, followed by 3 h of reperfusion (I/R) without or with capsaicin denervation and intravenous (i.v.) pretreatment with adiponectin (20 µg/kg), or a combination of CGRP (10 µg/kg s.c. = subcutaneously) and adiponectin (20 µg/kg i.v.). Results are mean ± SEM of 8–10 rats. Asterisk (*) indicates significant change compared with the respective values obtained in rats exposed to I/R alone (placebo group, VEH = vehicle). Cross (+) indicates significant change compared with the respective values obtained in rats exposed to VEH + I/R. Asterisk and cross (*+) indicate a significant change compared with the value obtained in rats with capsaicin denervation pretreated with adiponectin and exposed to I/R. Double cross (++) indicates a significant change compared to respective values obtained in rats with capsaicin denervation pretreated with adiponectin and exposed to I/R.

In the vehicle-pretreated control rats exposed to I/R, mucosal damage, as reflected by the squamation of the surface epithelium of the oxyntic mucosa as well as vascular congestion and micro bleeds, is observed (Figure 4B). In a rat pretreated with adiponectin (20 µg/kg) and subsequently exposed to I/R, the epithelial damage was superficial and mild desquamation of surface epithelium was observed, but the epithelial cellular lining and deeper layers of the glandular mucosa were preserved, indicating partial protection of the mucosal structure (Figure 4C). In the gastric mucosa of an animal in which L-NNA (20 mg/kg) was given with combination with adiponectin (20 µg/kg), the gastric epithelium was severely distorted (Figure 4D). This abnormal histopathological pattern observed in the gastric mucosae in the case of L-NNA pretreatment was improved when L-arginine (200 mg/kg) was combined with L-NNA (20 mg/kg) and adiponectin (20 µg/kg), as presented by a partial restoration of the glandular structure (Figure 4E vs. Figure 4D). As shown in Figure 5, the exposure of vehicle control animals to I/R produced numerous acute gastric mucosal lesions and GBF was significantly diminished, as compared to that in intact mucosae. Pretreatment with adiponectin (20 µg/kg i.v.) significantly reduced the gastric lesion area and raised GBF compared to vehicle control rats exposed to I/R. As shown in Figure 5, the functional ablation of sensory nerves by neurotoxic doses of capsaicin significantly augmented I/R-induced gastric lesions and this effect was accompanied by a significant decrease of GBF compared with that obtained in vehicle-pretreated I/R rats with intact sensory nerves (*p* < 0.05).

Administration of adiponectin (20 µg/kg i.v.) caused a significant decrease in I/R gastric lesion area and a significant increase in GBF (*p* < 0.05), compared with the respective values obtained in animals with functional ablation of sensory nerves, though these changes were less pronounced those recorded in animals with intact sensory nerves (Figure 5). The cotreatment of CGRP applied subcutaneously (s.c.) at a dose of 10 µg/kg with adiponectin (20 µg/kg i.v.) significantly decreased the area of gastric lesions and significantly raised GBF compared with those pretreated with adiponectin with and without deactivated sensory fibers (*p* < 0.05).

**Figure 6:**
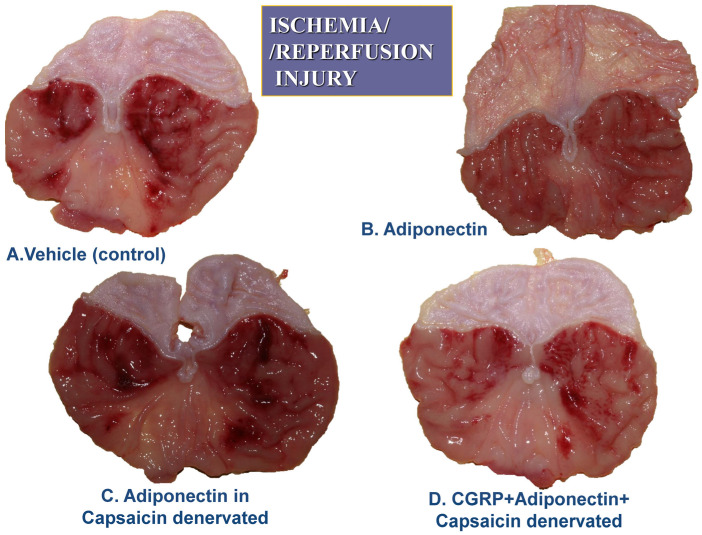
(**A**–**D**). The representative appearance of the rat pretreated with vehicle (control) and 30 min later exposed to 30 min of ischemia, followed by 3 h of reperfusion (I/R) shows the presence of multiple lesions observed in the oxyntic part of the gastric mucosa (**A**). In a rat pretreated with adiponectin (20 µg/kg) and subsequently exposed to I/R, the number of gastric mucosal erosions is reduced (**B**). The number of I/R-induced gastric lesions is increased in the gastric mucosae of animals with capsaicin denervation given in combination with adiponectin (20 µg/kg) (**C**), compared with that assessed in animals wherein adiponectin was applied alone in the I/R model [Adiponectin]. The number of I/R-induced gastric mucosal erosions was significantly diminished in gastric mucosae pretreated with CGRP (10 µg/kg), capsaicin denervation, and adiponectin (20 µg/kg) (**D**).

Figure 6 exhibits the representative appearance of a rat pretreated with vehicle (control) and 30 min later exposed to 30 min of ischemia followed by 3 h of reperfusion (I/R) (A). Similarly, as in Figure 3A, the gastric mucosa of a vehicle-pretreated animal exposed to I/R 30 min later shows the presence of multiple lesions observed in the oxyntic part of the gastric mucosa (Figure 6A). In a rat pretreated with adiponectin (20 µg/kg) and subsequently exposed to I/R, the severity of gastric mucosal erosions was reduced (Figure 6B). The area of I/R-induced gastric lesions is markedly increased in the gastric mucosae of capsaicin-denervated animals pretreated with adiponectin (20 µg/kg) and exposed to gastric I/R (Figure 6C) compared with that presented when adiponectin was applied to rats with intact sensory nerves exposed to I/R (Figure 6C vs. Figure 6B). The number and area of I/R-induced gastric mucosal erosions was diminished in the gastric mucosae of capsaicin-denervated rats pretreated with a combination of CGRP (10 µg/kg) and adiponectin (20 µg/kg) (Figure 6D vs. Figure 6C).

### 2.3. Lipid Peroxidation Products and Antioxidative Enzyme Activity

**Figure 7:**
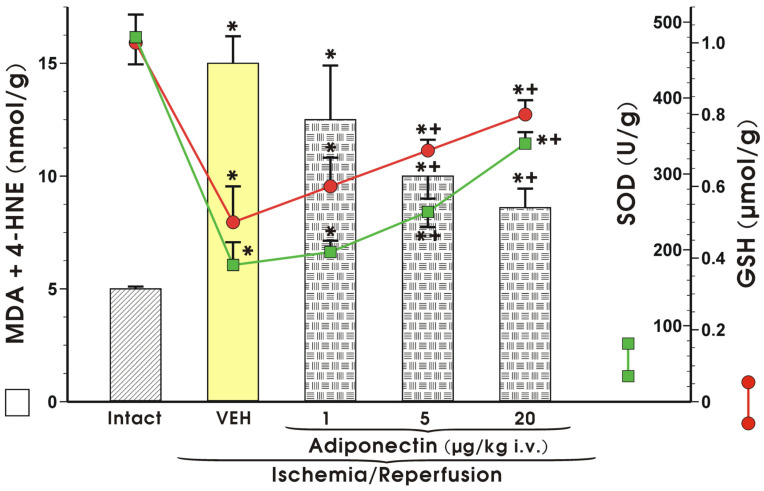
Concentration (nmol/g) of malondialdehyde (MDA) and 4-hydroxynonenal (4-HNE) and activity of superoxide dismutase (SOD) and reduced glutathione (GSH) in the gastric mucosae of rats exposed to 30 min of ischemia, followed by 3 h of reperfusion (I/R) without or with intravenous (i.v.) pretreatment with adiponectin, applied in graded doses, from 1 to 20 µg/kg. Results are mean ± SEM of 8–10 rats. Asterisk (*) indicates a significant change compared with the value obtained in intact rats (placebo group, VEH = vehicle). Asterisk and cross (*+) indicate a significant change compared with the value obtained in rats pretreated with adiponectin given at a dose of 1 µg/kg (i.v.) and exposed to I/R.

The concentration of MDA and 4-HNE in the intact mucosae (control) was very low and almost at the analytical limit of detection, but in vehicle-pretreated rats exposed to I/R, the level of MDA and 4-HNE significantly increased compared with those in the intact mucosae (*p* < 0.05) (Figure 7). Pretreatment with adiponectin (1 µg/kg i.v.) had no effect on MDA+4-HNE content, but when adiponectin was administered in a higher dose of 5 µg/kg i.v., a significant decrease in MDA and 4-HNE content compared to the values of the lipid peroxidation products was detected after I/R (*p* < 0.05) (Figure 7). Administration of a higher dose of adiponectin (20 µg/kg i.v.) led to a further significant decrease in concentration of these lipid peroxidation products (*p* < 0.05) (Figure 7).

SOD activity and GSH content reached the highest values in intact gastric mucosae (Figure 7). Following exposure to I/R in vehicle-pretreated rats, a significant decrease in SOD activity and GSH content were observed (*p* < 0.05) (Figure 7). Pretreatment with adiponectin (5 µg/kg i.v.) evoked a significant increase in SOD activity and GSH concentration, as compared to the values of these antioxidative factors detected in vehicle-pretreated rats exposed to I/R. Administration of higher dose of adiponectin (20 µg/kg i.v.) led to a significant increase in the mucosal concentration of GSH and SOD activity compared to the respective mucosal concentrations of GSH and SOD observed in vehicle-pretreated control rats and to those pretreated with adiponectin at a dose of 1 µg/kg (*p* < 0.05) (Figure 7).

### 2.4. Expression of Interleukin-1 Beta (IL-1β) and Tumor Necrosis Factor Alpha (TNF-α) mRNA in the Gastric Mucosa

**Figure 8:**
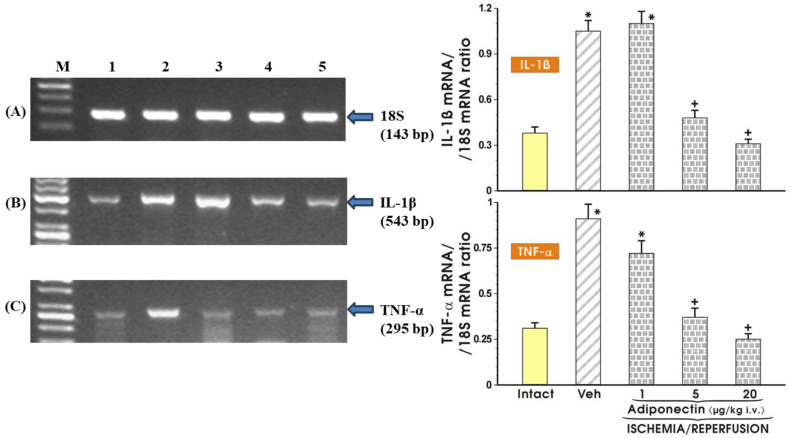
The messenger RNA expression for interleukin 1-beta (IL-1β) (panel (**B**)) or tumor necrosis factor alpha (TNF-α) (panel (**C**)) analyzed by reverse transcriptase PCR and compared with reference housekeeping gene 18S (panel (**A**)) in the intact gastric mucosae and in rats exposed to ischemia (30 min) followed by 3 h of reperfusion (I/R) without or with intravenous (i.v.) pretreatment with adiponectin, applied in graded doses from 1 to 20 µg/kg. Results are mean ± SEM of 8–10 rats. Asterisk (*) indicates a significant change compared with the values obtained in intact gastric mucosae (intact). Cross (+) indicates a significant change compared with the value obtained in rats exposed to I/R alone (Veh).

Figure 8 shows the data with an RT PCR analysis of mRNA expression for IL-1β and TNF-α, respectively, in the gastric mucosae of intact rats and those pretreated with graded doses of adiponectin ranging from 1 µg/kg up to 20 µg/kg against the gastric lesions induced by I/R. The IL-1β and TNF-α mRNA were weakly detectable in the intact gastric mucosa. In contrast, a strong signal for IL-1β and TNF-α mRNA in vehicle-treated rats exposed to I/R (Veh) was detected. The analysis of the signal expression for IL-1β and TNF-α revealed that the gastric mucosal expression of IL-1β and TNF-α mRNA was significantly decreased in rats pretreated with graded doses of adiponectin (1–20 µg/kg i.v.) compared with vehicle control rats exposed to I/R.

## 3. Discussion

This study has been designed to determine whether adiponectin, one of the most potent adipokines [10], can afford gastroprotection against gastric lesions induced by I/R. Our second goal was to examine the possible mechanism of adiponectin’s protective action against gastric I/R damage. Previous studies focused on adiponectin-induced protection against ischemic changes in the heart, especially under diabetic conditions [11,12,13]. These authors have documented the beneficial, cardioprotective action of adiponectin, involving antioxidative and reactive oxygen metabolite scavenging mechanisms [14,15,16]. Previous studies revealed that adiponectin can affect the course of experimental colitis in obese mice subjected to voluntary exercise [17,18,19,20]. In the case of the stomach, the beneficial role of adiponectin in gastric cancer pathogenesis has been emphasized [21,22,23,24]. However, the mechanism of protective action of adiponectin in the pathogenesis of gastric damage has been little studied. Kemmerly et al. [25] have suggested that adiponectin may play an important role in the mechanism of gastrointestinal mucosal defense, involving attenuation of neutrophil infiltration, enhancement of mucin synthesis, and cooperation of this peptide with nitric oxide (NO), hydrogen sulfide (H_2_S) and the antioxidizing enzyme heme oxygenase (HO). Yamamoto et al. [26] have shown protective action of exogenous administration of adiponectin against ethanol-induced gastric damage, using adiponectin-knockout mice, but the potential protective effect of adiponectin in an acid-dependent model of acute gastric damage such as that evoked by I/R has not been studied. Herein, we documented that parenteral application of adiponectin exhibited dose-dependent protective action against I/R-induced gastric mucosal damage and this protection was accompanied by an increase in GBF. This enhancement in the blood flow prompted us to explore the involvement of NO in this protection, since NO is a crucial factor involved in regulation of gastric mucosal microcirculation [6,7]. This is why we employed the N-nitro-L-arginine (L-NNA) to inhibit NO synthase with and without the combination of L-arginine, the precursor of NO bioactivity. We found that the NOS inhibition impeded the protective action of adiponectin against the I/R conditions of the stomach. Antagonism between the beneficial action of adiponectin and NOS blockade was also evidenced in a kidney [27,28]. Ndisang et al. [27] showed that inhibition of NOS by treatment with nitric L-arginine derivative counteracted the hypotensive action of adiponectin, leading to hypertension. Furthermore, it has been shown that the beneficial action of adiponectin on gastric mucosa was restored after the combined treatment of this adipokine with L-arginine, a substrate for NOS, and L-NNA. These results are in accordance with other investigations, which documented synergistic interplay between L-arginine supplementation and the action of adiponectin in other organs [29,30,31,32]. In contrast, when D-arginine was combined with L-NNA instead of L-arginine, the reversal of the protective action of adiponectin by L-NNA was still evident. Interestingly, the increase in NO production via the AMP-associated metabolic pathway was required for the protective action of adiponectin against cardiac ischemia and reperfusion [33].

Our study revealed for the first time that capsaicin-sensitive afferent neurons can be involved in the gastroprotective action of adiponectin. This notion is based on our finding that capsaicin-induced functional ablation of sensory nerves completely reversed the beneficial action of adiponectin against gastric damage induced by I/R. Kentish et al. [34] have also implicated the role of the neural vagal component in the metabolic action of adiponectin, but the role of capsaicin-sensitive afferent fibers in gastroprotection was not closely investigated. We have observed that supplementation of CGRP (sensory nerve neuromediator) restored the protective action of adiponectin against gastric lesions induced by I/R in capsaicin-denervated rats. Abu Bakar et al. [35] reported on the importance of sensory nerves and CGRP signaling in the pathomechanism of perivascular adipose tissue-induced vasodilatation and leptin release. Vasoconstriction resulting from the destruction of these nerves was associated with the fall in the level of adiponectin in tissues [35].

The possibility that adiponectin can attenuate I/R-induced oxidative stress has not been fully explored. Therefore, we examined the participation of adiponectin in the mechanism of lipid peroxidation products MDA and 4-HNE in adiponectin-induced gastroprotection against I/R injury. We have documented that adiponectin applied in graded doses attenuated the process of lipid peroxidation, which was manifested by a decrease in MDA and 4-HNE in gastric mucosa injured by I/R. These gastric mucosal changes were counteracted by adiponectin in a dose-dependent manner suggesting that gastroprotection by adiponectin depends, at least in part, on its antioxidative properties. In fact, Liu et al. [36] found that adiponectin can reduce gastric mucosal I/R injury by a decrease in MDA content. This antioxidative action of adiponectin could be linked with a maintenance of NO at low physiological levels by this adipokine, because L-NNA counteracted the adiponectin-induced protection and accompanying hyperemia in gastric mucosae compromised by I/R. In keeping with this notion is the observation that adiponectin can inhibit oxidative nitrative stress, thus preventing cardiac damage associated with myocardial infarction [37].

The next aim of our study was to determine effect of adiponectin on gastric acid secretion. We have evidenced that administration of adiponectin dose-dependently reduces gastric acid secretion with an accompanied increase in gastrin plasma levels. Gastric acid is a corrosive substance and our previous studies confirmed that the inhibition of gastric acid secretion diminished the risk of gastric mucosal barrier destruction [38]. An accompanying rise in gastrin may suggest that adiponectin inhibited the secretory activity of parietal cells in gastric glands, which in turn caused hypochlorhydria and enhanced gastrin release [39].

Our study documented the protective effect of adiponectin application related to a reduction in IL-1β and TNF-α expression. Varol et al. [40], in a diet-induced obesity model, confirmed that a reduction in key pro-inflammatory cytokines, such as IL-1β and TNF-α, was related to an increase in adiponectin, and revealed a beneficial effect in that model. Liu and other contributors [1] evidenced, in a myocardial ischemic model, that TNF-α blockade increased adiponectin production, leading to decrement of oxidative stress. Our model also exhibited the beneficial, gastroprotective effects of pro-inflammatory cytokine decrease and an accompanying reduction of oxidative stress after adiponectin administration. Burgess et al. [41] suggested that the inducible form of heme oxygenase (HO-1) was involved in the interplay between TNF-α and adiponectin. Namely, up-regulation of HO-1 decreased the production of TNF-α and increased the level of adiponectin.

In summary, we conclude that adiponectin administered intravenously exerted a gastroprotective effect against I/R, through mechanisms involving a decrease in lipid peroxidation (MDA+4-HNE) and gastric acid secretion, as well as an increase in GBF mediated by endogenous NO and capsaicin-sensitive afferent nerves.

## 4. Material and Methods

### 4.1. Animals

Experiments were carried out on 160 male Wistar rats, weighing about 200 g and fasted for 24 h before all studies. Studies were approved by the Ethic Committee for Animal Research of Jagiellonian University College of Medicine (No. 923/2024) in Krakow, Poland (28 November 2024), compliant with international guidelines (EU Directive 2010/63).

### 4.2. Gastric Secretory Studies

To determine the effect of adiponectin on gastric acid secretion in separate series, rats were equipped with gastric fistulas about 2 weeks before gastric juice collection. On the day of investigations, the fistula was opened and the stomach was rinsed gently with 5–20 mL of tap water at 37 °C. The basal gastric secretion was collected for 60 min and then adiponectin (1–40 µg/kg) was applied (i.v.). In the control group, vehicle—saline—was administered and the volume of the gastric juice and the gastric concentration of H^+^ were determined using an automatic titrator (Radiometer, Copenhagen, Denmark) for each sample collected for 30 min and expressed as mean outputs per 30 min [38]. The scheme of the gastric secretion experiments was as follows:

Time: 0;

1st fraction (30 min later)—basal gastric secretion;

2nd fraction (30 min after 1st fraction)—basal gastric secretion;

Administration of adiponectin—investigated or vehicle (saline);

3rd fraction (30 min later);

4th fraction (30 min after 3rd);

5th fraction (30 min after 4th);

6th fraction (30 min after 5th, respectively).

For group A (gastric secretion) n (number of animals) = 8 for each group → Σn = 40 (8 rats × 5 groups: vehicle, adiponectin 1 µg/kg, adiponectin 5 µg/kg, adiponectin 20 µg/kg, adiponectin 40 µg/kg).

### 4.3. Production of Gastric Lesions

In group B, only vehicle, i.e., saline, was used and the animals did not undergo any procedures (n = 8). In group C, gastric lesions were induced by ischemia followed by reperfusion (I/R) (n = 8). Briefly, a celiac artery was identified and clamped for 30 min (I). Followed by the release of this clamp, the flow through the celiac artery continued for 3 h (R), as proposed by Wada et al. and our group [5,6,42]. In group D, adiponectin (Abcam, Cambridge, UK) was applied intravenously (i.v.) in gradually increasing doses, namely 1 µg/kg (n = 8), 5 µg/kg (n = 8), 20 µg/kg (n = 8) and 40 µg/kg (n = 8) 30 min prior to the start of I/R. In group E, N^G^-nitro-L-arginine (L-NNA) (Merck, Darmstadt, Germany) was applied intraperitoneally (20 mg/kg i.p.) with (n = 8) or without (n = 8) adiponectin (20 µg/kg i.v.) prior to 3 h of I/R. Group F was pretreated with L-arginine (Merck, Darmstadt, Germany) (n = 10) or D-arginine (Merck, Darmstadt, Germany) (n = 8), applied intragastrically (i.g.) in a dose of 200 mg/kg followed 30 min later by L-NNA (20 mg/kg i.p.), administrated adiponectin (20 µg/kg i.v.), and then subsequently exposed to I/R. In group G, consisting of capsaicin-denervated rats, vehicle (n = 8) or adiponectin (20 µg/kg i.v.) was administered with (n = 10) or without (n = 8) calcitonin gene-related peptide (CGRP) (Abcam, Cambridge, UK), applied at a dose of 10 µg/kg (s.c.). For this purpose, the animals were pretreated with capsaicin (Sigma Co., St. Louis, MO, USA) injected subcutaneously (s.c.) for 3 consecutive days at a dose of 25, 50 and 50 mg/kg (total of 125 mg/kg) about 2 weeks before the experiment. All injections of capsaicin were performed under ether anesthesia to counteract the pain reaction and respiratory impairment associated with the injection of this agent. To check the effectiveness of the capsaicin denervation, a drop of 0.1 mg/mL solution of capsaicin was instilled into the eye of each rat and the protective wiping movement was counted, as described previously [5,43].

### 4.4. Determination of Gastric Blood Flow and Area of Gastric Mucosal Lesions

The evaluation of gastric lesions and gastric blood flow (GBF) was performed at the end of 3 h of I/R. To measure GBF, a laser Doppler flowmeter (Laserflo, model BPM 403A, Blood Perfusion Monitor, Vasamedics, St. Paul, MN, USA) was employed. The animals were anesthetized with 2% isoflurane, then the abdomen was opened and the stomach was exposed to determine the GBF. GBF was measured on the anterior and posterior walls of the stomach not containing gastric lesions. The mean values of three measurements were calculated and expressed as a percentage change from the value recorded in the intact mucosa. The area of gastric lesions was determined by computerized planimetry (Morphomat, Carl Zeiss, Berlin, Germany), as described previously [5,6].

### 4.5. Measurement of Lipid Peroxidation

The determination of the levels of malondialdehyde (MDA) was carried out and their levels were used as indicators of lipid peroxidation [5]. For the determination of MDA and 4-HNE, about 600 mg of gastric mucosa was excised. Then, 20 µL 0.5 M BHT (butylated hydroxytoluene) was added to prevent sample oxidation. This sample was subsequently homogenized in 20 mM Tris for 15 s in pH = 7.4. Then, the homogenate was centrifuged (3000× *g* at 4 °C for 10 min) and the obtained clear supernatant was stored at −80 °C until testing.

The colorimetric assay for lipid peroxidation (Bioxytech LPO-586, Oxis, Portland, OR, USA) was used to determine the concentrations MDA and 4-HNE in the tissue. This assay is based on the reaction of a chromogenic reagent N-methyl-2-phenylindole with MDA and 4-HNE at 45 °C. This reaction yields a stable chromophore with maximal absorbance at 586 nm. This absorbance was measured by a Marcel s300 (Marcel S.A., Warsaw, Poland) spectrophotometer. The results were expressed as nanomoles per gram of tissue (nmol/g) [5,6].

### 4.6. Determination of Superoxide Dismutase (SOD) Activity

To determine the activity of SOD, a sample of gastric mucosa was obtained, as described above. The colorimetric assay for assessment of SOD activity (Bioxytech SOD-525, Oxis, Portland, OR, USA) was used. This method is based on the SOD-mediated increase in the rate of autooxidation of tetrahydrobenzofluorene in aqueous alkaline solution to yield a chromophore with maximum absorbance at 525 nm. This absorbance was measured by a Marcel s300 (Warsaw, Poland) spectrophotometer and the results were expressed as units per gram of gastric tissue (U/g) [5,6].

### 4.7. Measurement of Gastric Mucosal Tissue Glutathione Level (GSH) and Plasma Gastrin Concentration

To determine the level of the reduced form of glutathione (GSH), a sample of gastric mucosa was taken, as described above. Then, a 5% aqueous solution of metaphosphoric acid was added to evoke protein precipitation. Then, a colorimetric assay for the assessment of reduced glutathione concentration (Bioxytech, GSH-400, Oxis, Portland, OR, USA) was used. The level of reduced glutathione was measured, with the maximal absorbance at 400 nm, by a Marcel s300 spectrophotometer (Marcel S.A., Warsaw, Poland). Results were expressed as micromole per gram of tissue (μmol/g) [5,6].

Immediately after GBF measurement, about 2 mL of venous blood sample was withdrawn from the vena cava into EDTA-containing vials and used for the determination of plasma gastrin levels by radioimmunoassay (RIA), as described previously [44].

### 4.8. Determination of Mucosal IL-1β and TNF-α mRNA Using Reverse Transcriptase Polymerase Chain Reaction (PCR)

Reverse transcriptase PCR was performed as described previously [6]. Samples of gastric mucosa, weighing about 500 mg, were scraped on ice using glass slides and then immediately frozen in liquid nitrogen and stored at a temperature of −80 °C. The total RNA was isolated from the mucosa according to the method of Chomczynski and Sacchi [45], using a rapid guanidinium isothiocyanate/phenol chloroform single-step extraction (Heidelberg, Germany). After precipitation, the RNA was resuspended in RNase-free Tris EDTA buffer, and the concentration was estimated by absorbance at 260 nm wavelength. Samples were frozen at −80 °C until analysis.

First-strand cDNA was synthesized from the total cellular RNA (5 µg) using 200 U Strata Script RT (Stratagene, La Jolla, CA, USA). After the reverse transcription, the transcriptase activity was destroyed by heating, then the cDNA was stored at −20 °C until PCR.

Single-stranded cDNA was generated from 5 µg total cellular RNA, using Moloney murine leukemia virus reverse transcriptase (MMLV-RT) and oligo-(dT) primers. The PCR mixture was amplified in a DNA thermal cycler (Thermo Fisher Scientific, Waltham, MA, USA). The primers were synthesized by Gibco BRL/Life Technologies (Eggenstein, Germany). The PCR products were detected by electrophoresis on 1.5% agarose gel, containing ethidium bromide. The location of the predicted products was confirmed using a 100 bp ladder (Takara, Shiga, Japan) as a standard size marker.

### 4.9. Statistical Analysis

The experiments and data collection were done by operators blinded to the sample identity. The results are expressed as means ± SEM. Statistical analysis was done using nonparametric tests: (1) A Mann–Whitney test to analyze differences between two investigated groups; (2) for analyses with more than two experimental groups, the data were analyzed by a Kruskal–Wallis test, followed by Dunn’s multiple comparison post hoc test, taking into account the correction for multiple comparisons, which involved reducing the nominal significance level for each of a set of related tests, directly proportional to their total number. The Bonferroni test was used as a correction method, and a family of tests has been constituted per figure. Differences with *p* < 0.05 were considered significant.

Randomization has been done using random number tables. The final *n* per group was an average of 8–10 rats. The sample size has been established based on our *prior* experience in these types of experiments. Our previous studies [5,6] have documented that a larger sized group of animals (n = 10) used with the combined treatment of many substances would result in more reliable outcomes.

For gastric fistula experiments with serial sampling, each animal outcome was summarized and calculated as mean post-dose output over a defined period ± standard error of the mean (SEM) and the results of particular groups were compared with the vehicle group using a Mann–Whitney test with multiplicity adjustment. Baseline secretion was handled, including the two 30 min fractions 1 and 2 (1 h period). The exact post-dose time to compute per-animal means was similar in fractions number 3–6 (2 h after substance administration). Comparisons were performed for each dose vs. vehicle with multiplicity correction.

## 5. Conclusions

The present study provides evidence that adiponectin plays a beneficial role in the experimental model of gastric damage induced by I/R. Adiponectin dose-dependently decreased gastric I/R lesions. Adiponectin inhibits gastric acid secretion. The expression of mRNA for pro-inflammatory cytokines is diminished after adiponectin administration, confirming its antioxidative properties. Adiponectin affects oxygen metabolism during I/R by a reduction in lipid peroxidation (MDA+4-HNE, as indicators of oxidative damage) and strengthening of tissue-scavenging properties (SOD and GSH). The release of NO by afferent C fibers is a necessary condition for the beneficial effect of adiponectin during I/R (destruction of these fibers abolishes the protective effect during I/R, restored by supplementation of CGRP). NO serves as mediator for adiponectin action in the I/R model, which is evidenced by the blockade of NO synthesis by L-NNA and this effect is restored by L-arginine administration. These results reveal the role of adiponectin in acute gastric damage healing by antioxidative, antisecretory and anti-inflammatory activity, mediated by NO.

## Figures and Tables

**Table 1:** Gastric acid output (µmol/30 min) in rats equipped with gastric fistulas, without or with intravenous (i.v.) pretreatment with adiponectin, applied in graded doses, from 1 to 40 µg/kg. Results are mean ± SEM of 8–10 rats. Asterisk (*) indicates a significant change compared with the value obtained in intact rats (without treatment with adiponectin).

Group of the Investigated Animals	Acid Output (µmol/30 min)
Vehicle	85 ± 16
Adiponectin 1 µg/kg (i.v.)	80 ± 11
Adiponectin 5 µg/kg (i.v.)	68 ± 10 *
Adiponectin 20 µg/kg (i.v.)	53 ± 9 *
Adiponectin 40 µg/kg (i.v.)	48 ± 12 *

## Data Availability

The data that support the findings of this study are available from the corresponding author upon reasonable request.
